# Trend of Sudden Unexpected Death in Epilepsy Incidence Rate in Rural West China

**DOI:** 10.3389/fneur.2021.735201

**Published:** 2021-09-24

**Authors:** Haijiao Wang, Deng Chen, Jun He, Yujin He, Ling Liu, Dong Zhou

**Affiliations:** ^1^Department of Neurology, West China Hospital, Sichuan University, Chengdu, China; ^2^Sichuan Center of Disease Control and Prevention, Chengdu, China

**Keywords:** sudden death, epilepsy, trend, rural, incidence rate

## Abstract

**Objectives:** To explore the trend of sudden unexpected death in epilepsy (SUDEP) incidence rate over time in rural west China.

**Methods:** We scanned probable SUDEP patients from the epilepsy program between 2010 and 2019 in rural West China and performed a verbal autopsy for each eligible patient. We calculated the crude and sex-adjusted incidence rate of SUDEP per person-year over a calendar year and the year of follow-up. We calculated the incidence rate ratio with the Poisson model in STATA 12.0 and calculated the annual percentage change (APC) and average annual percentage change in Joinpoint Trend Analysis Software 4.8.0.1 to analyze the trend of SUDEP incidence rate.

**Results:** In 2010–2019, 44 probable SUDEPs were identified from 10,128 patients with a total person-year of 31,347. The crude and sex-adjusted incidence rates of SUDEP were 1.40 and 1.45%0. Twenty-five (56.8%) of the 44 probable SUDEPs had no generalized tonic-clonic seizure 3 months before their death. The incidence of probable SUDEP decreased significantly in the calendar year [APC = −11.7, 95% confidence interval (CI): −21.7 to −0.3] and in time of follow-up (average annual percentage change = −21.2, 95% CI: −34.3 to −5.4). Comparing the first 5 years in follow-up with the subsequent 3 years, the incidence rate of SUDEP decreased significantly (estimated incidence rate ratio = 0.4, 95% CI: 0.2 to 0.8).

**Significance:** SUDEP happened to 1.4 cases per thousand patient-years in convulsive epilepsy in rural west China between 2010 and 2019. The incidence rate of SUDEP presented a downward trend over the time of follow-up.

## Introduction

Sudden unexpected death in epilepsy (SUDEP) is the leading cause of mortality in patients with epilepsy (PWE), estimated to be 1.2 SUDEP cases per 1,000 PWE in a meta-analysis of population-based studies (1). In a recent study of the vagus nerve stimulator, Ryvlin reported that the incidence rate of SUDEP decreased significantly from 2.5 to 1.7 per 1,000 person-years with a 10-year follow-up in refractory PWE (2). Similarly, Tomson showed that the incidence rate of SUDEP decreased by 7% per year with a 6-year follow-up in Sweden (3). In the United States, the trend of the SUDEP incidence rate also decreased from 2009 to 2016 (4). Several studies have reported a decreasing trend of SUDEP in some developed countries (2, 3, 5), but it is unknown in developing countries. However, the incidence of SUDEP is higher in patients with lower socioeconomic disparities (6), probably because of the lack of specialty care or lower knowledge and awareness of epilepsy (7). A Chinese community-based cohort showed that the incidence of probable SUDEP was 2.0 per 1,000 person-years, which is higher than the rate in high-income countries (8). In rural West China, our team previously reported that the incidence of probable SUDEP was 1.7 cases per thousand patient-years in people with convulsive epilepsy in 2015 (9). It is unknown whether the SUDEP incidence rate is decreasing in low-income countries. This article aims to reveal SUDEP incidence rate trends in rural western China.

## Materials and Methods

### Study Population

This study was approved by the Sichuan University Ethical Standards Committee on Human Experimentation.

All patients were obtained from the epilepsy management program at the primary healthcare level in 10 counties in Sichuan Province, rural West China, from 2010 to 2019. All participants gave informed consent before inclusion in the program. The background and operation procedures of this program have been reported in a previous study. Patients in the program were adults diagnosed with generalized tonic-clonic seizure (GTCS) epilepsy and treated with phenobarbital. Enrolled patients visited trained physicians every 2 weeks for the first 2 months and monthly after that. At each visit, physicians adjusted the dose when necessary and filled in a follow-up form that recorded numbers of convulsive seizures and adverse events experienced by the patients and checked adherence by counting residual tablets. A patient was considered to have poor medication compliance if they had taken more or less than a 3-day dose of medication within 4 weeks on 3 consecutive occasions (10).

First, according to the unified death record form, we excluded patients with definite causes of death, such as accidents, drowning, and physical diseases. We performed a verbal autopsy (11) for each eligible patient with an unknown cause of death by directly connecting the first witness and interviewing the relatives and friends of the deceased to identify the probable SUDEP. The interview sought key symptoms and signs through investigating the detail of death with a uniform list for investigation, especially included the circumstances of the death, whether the patients died during sleep at night or in the course of an epileptic seizure. The criteria of probable SUDEP were in accordance with the definition of Nashef (12)—the epilepsy patient was found dead in benign circumstances, with no other relevant pre-existing conditions, but without post-mortem examination. We examined our records to ensure that individual data were not repeatedly entered in the analysis. All patients participated voluntarily and signed written notice of consent.

For each probable SUDEP, we extracted the age, sex, height, weight, follow-up records, dose of phenobarbital, age of seizure onset, duration of epilepsy, drug combination, and seizure frequency in the past 3 months. Due to the different times of the patients entering the program, we used the person-year to calculate the incidence rate. We calculated the crude incidence rate of SUDEP per person-year over calendar years. At the same time, we calculated the incidence rate of SUDEP over the year of follow-up according to the follow-up records of each patient. To explore the trend of the SUDEP rate, we split the 10-year study period equally into two different groups: 2010–2014 and 2015–2019.

### Statistical Analysis

We analyzed the crude incidence rate and sex-adjusted incidence rate of SUDEP according to the sex ratio from the Chinese Sixth National Census in 2010 (13).

Stata 12.0 and Joinpoint 4.8.0.1 were used for statistical analysis. We applied Joinpoint Trend Analysis Software 4.8.0.1 (14) from the American National Cancer Institute Division of Cancer Control and Population to calculate the annual percentage change (APC) and average annual percentage change (AAPC), 95% confidence intervals (CIs), and *P*-values to analyze trends in the SUDEP incidence rate. We compared the incidence rate ratio (IRR) of SUDEP during the last 3 years of follow-up against the first 5 years and during 2010–2014 against 2015–2019. We calculated the IRR with a Poisson model. Data with normal distributions are presented as the mean and variance, and other forms of data are presented as the median and quartile. For the outliers, we performed a sensitivity analysis by omitting it. We assigned the significance level as α = 0.05, with *p* < 0.05 considered statistically significant.

## Results

In 2010–2019, a total of 10,128 patients with 31,347 person-years were enrolled in the cohort of total follow-up. Among those, 44 probable SUDEP patients had been reported in the program, and the total crude incidence rate of probable SUDEP was 1.40 cases per thousand patient-years. The sex-adjusted incidence rate of SUDEP in 2010–2019 was 1.45%0 per person-year. The characteristics of the probable SUDEP patients are shown in Table 1. There were 56.8% (25/44) probable SUDEPs without GTCS 3 months before their death. The average GTCS frequency 3 months before death was 1.7 times.

**Table 1:** Characteristic of probable SUDEP in 2010–2019.

	**Total probable SUDEP (mean ± SD)**
Age	41.4 ± 15.9
Sex, female (%)	56.8%
Height (cm)	156.9 ± 7.8
Weight (kg)	52.0 (12.0)^†^
Average of follow up (years)	2.2 ± 3.5
Dosage of phenobarbital (mg)	90.0 (30.0)^†^
Age of onset (years)	18.9 ± 14.9
Duration of epilepsy (years)	24.6 ± 16.0
Combined medication (%)	15.9%
Poor medication compliance (%)	13.6%
Baseline seizure frequency of 1 year	12 (25)^†^
Last seizure frequency of 3 months before death	0 (3)^†^

† *Data were shown as median (quartile)*.

The dynamically changing incidence rate of probable SUDEP with the time of the calendar years 2010–2019 is shown in Table 2, and the time of follow-up is shown in Table 3. The longest follow-up of probable SUDEP patients in the management of our program was 7.4 years. Therefore, we calculated the incidence rate for each follow-up year over 8 years. The number of probable SUDEP patients in our study was not enough to analyze the age-adjusted incidence rate, but we analyzed the average age per year in 2010–2019 to explore the effect of age on the rate. We found that the age of patients increased throughout the year in the cohort.

**Table 2:** Trend of SUDEP incidence rate in 2010–2019.

**Year**	**Age of patients (mean ± SD)**	**Male**			**Female**			**Total**			
		**No. of SUDEP**	Person-year^*^	**Crude rate (%)**	**No. of SUDEP**	Person-year^*^	**Crude rate (%)**	**No. of SUDEP**	Person-year^*^	**Crude rate (%)**	**Sex-standardized rate of SUDEP (%)**
2010	38.10 ± 16.05	5	1,307	3.8	4	1,083	3.7	9	2,391	3.8	3.8
2011	38.54 ± 16.38	1	1,596	0.6	1	1,187	0.8	2	2,784	0.7	0.7
2012	39.24 ± 16.99	3	1,946	1.5	2	1,467	1.4	5	3,413	1.5	1.5
2013	39.94 ± 17.48	1	2,102	0.5	2	1,607	1.2	3	3,709	0.9	0.8
2014	40.68 ± 17.31	3	2,482	1.2	5	1,886	2.7	8	4,368	1.9	1.8
2015	41.69 ± 17.21	2	2,356	0.8	5	1,758	2.8	7	4,114	1.8	1.7
2016	41.97 ± 17.28	1	1,504	0.7	1	1,111	0.9	2	2,614	0.8	0.8
2017	43.08 ± 16.98	0	1,490	0.0	3	1,121	2.7	3	2,610	1.3	1.1
2018	44.69 ± 16.08	1	1,172	0.9	1	850	1.2	2	2,022	1.0	1.0
2019	44.94 ± 16.37	2	1,902	1.1	1	1,421	0.7	3	3,323	0.9	0.9
Total	41.36 ± 15.88	19	17,856	1.1	25	13,491	1.9	44	31,347	1.4	1.4

* *The person-year of each year was calculated by the number of follow-up record in each year. The total person-year was calculated by the total number of follow-up record in 2010-2019 rather than by summarizing of the each person-year*.

**Table 3:** Trend of SUDEP incidence rate with the time of follow-up.

**Time of follow-up**	**Number of patients**	**Number of SUDEP**	**Incidence rate (%)**
<1 year	10,128	44	4.3
1–2 years	6,967	36	5.2
2–3 years	4,879	23	4.7
3–4 years	3,666	18	4.9
4–5 years	3,139	13	4.1
5–6 years	2,382	8	3.4
6–7 years	1,800	2	1.1
7–8 years	1,219	1	0.8

**Figure 1:**
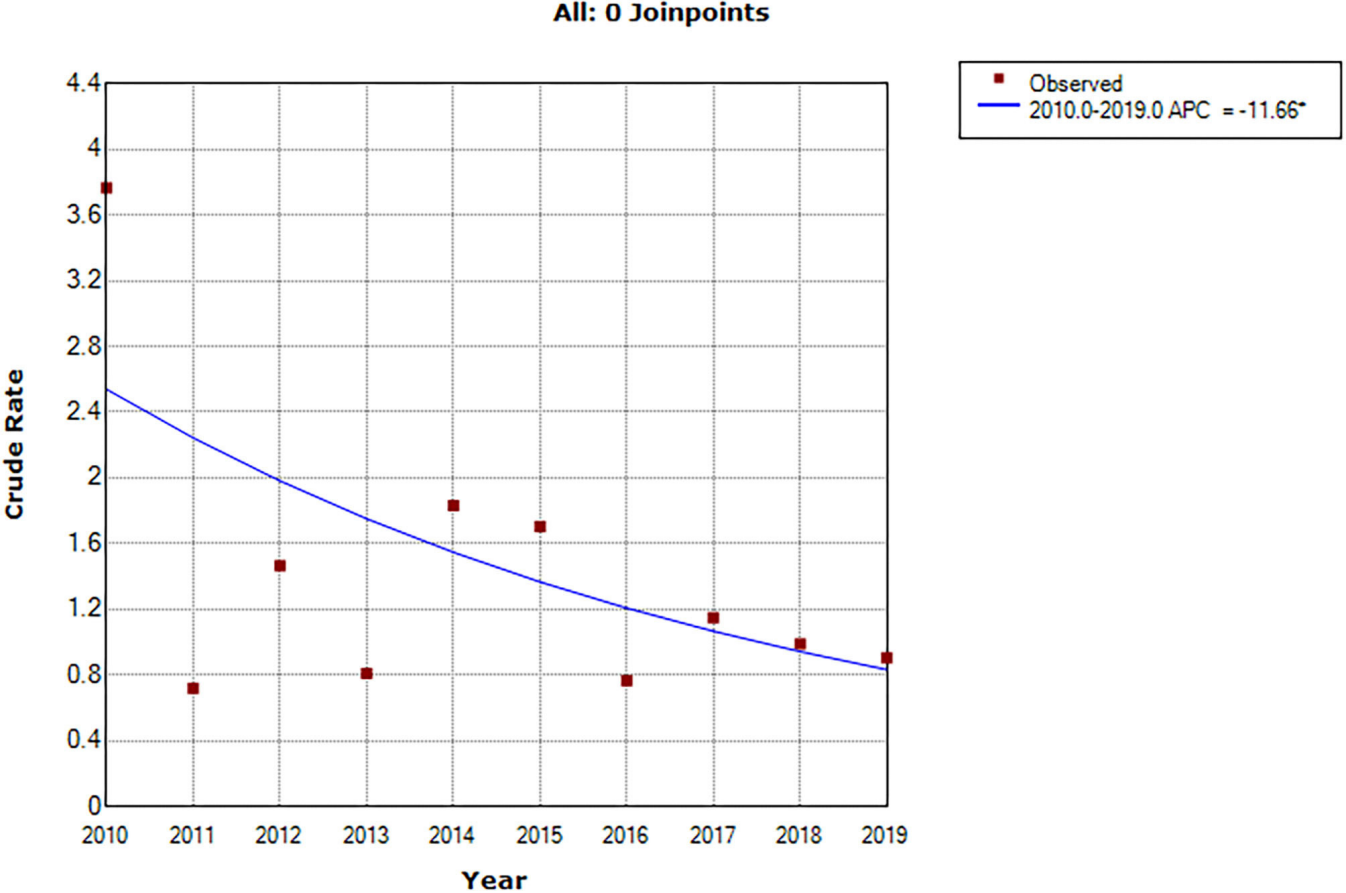
APC of crude SUDEP incidence rate in 2010–2019. *Indicates that the Annual Percent Change (APC) is significantly different from zero at the alpha = 0.05 level. Final Selected Model: 0 Joinpoints.

The incidence of probable SUDEP decreased significantly in 2010–2019 (APC = −11.7, 95% CI: −21.7 to −0.3, *P* = 0.045; Figure 1). The crude incidence rate of probable SUDEP in 2010–2014 was not significantly higher than the rate in 2015–2019 (estimated IRR = 0.7, 95% CI: 0.4–1.3, *P* = 0.3). The sex-adjusted incidence rate of probable SUDEP was also not significantly higher (estimated IRR = 0.7, 95% CI: 0.4–1.3, *P* = 0.3). The incidence rate of probable SUDEP in females seemed to be higher than that in males, but the difference was not significant (estimated IRR = 1.7, 95% CI: 0.96–3.15, *P* = 0.1).

**Figure 2:**
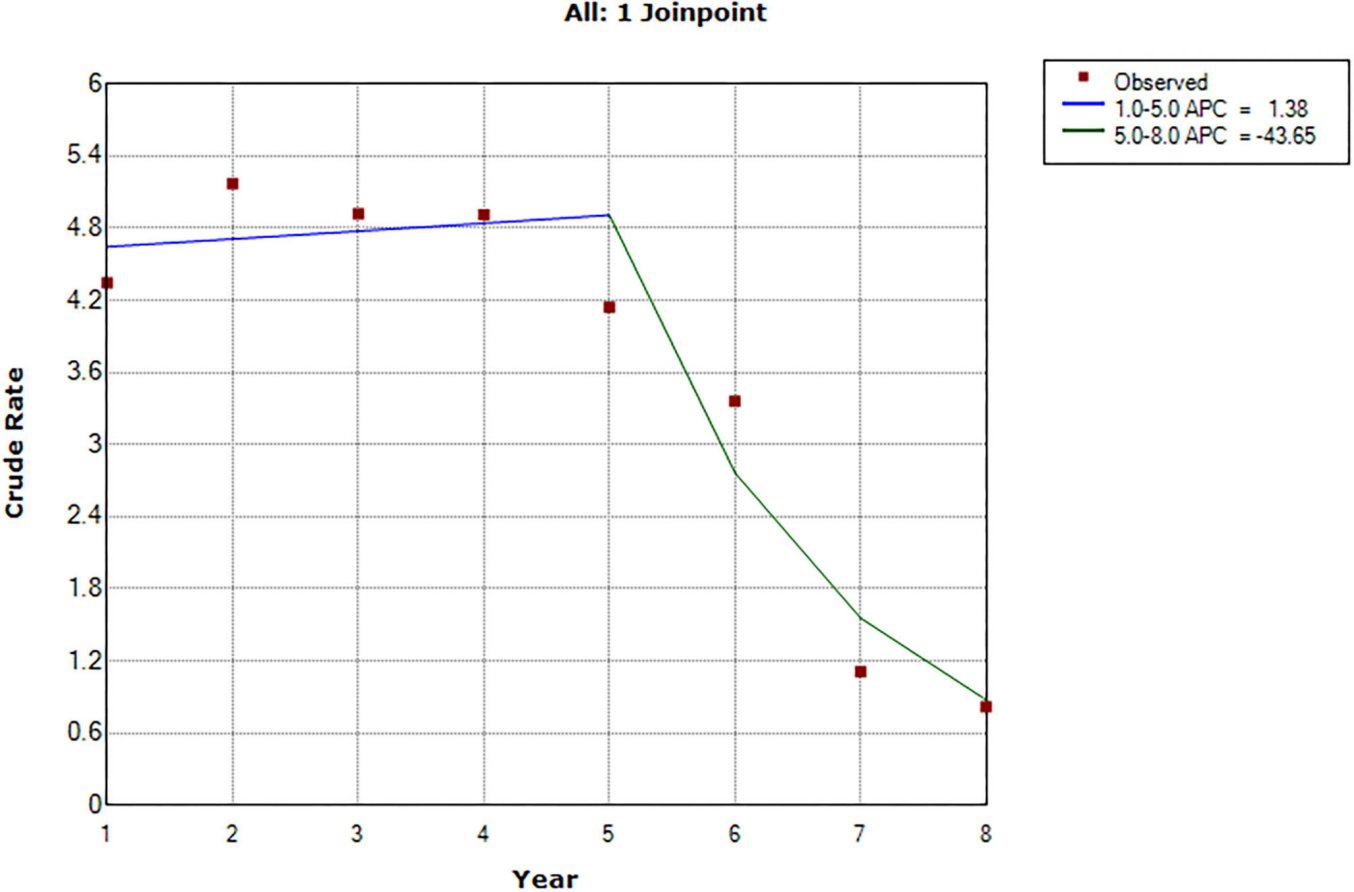
APC of crude SUDEP incidence rate after 1–8 years of follow-up. Final Selected Model: 1 Joinpoints.

Figure 2 shows one Joinpoint in the 5th year of follow-up. Within the first 5 years of follow-up, the crude rate of probable SUDEP increased slightly but not significantly (APC = 1.4, 95% CI:−10.3 to 14.5, P = 0.7; Figure 2). However, at follow-up between 5 and 8 years, the incidence rate of probable SUDEP had a decreasing trend (APC = −43.6, 95% CI: −71.2 to 10.3 *P* = 0.1; Figure 2). The average trend in the time of follow-up significantly decreased (AAPC = −21.2, 95% CI: −34.3 to −5.4, *P* = 0.01). Comparing the first 5 years with the subsequent 3 years in the follow-up of the program, the incidence rate of SUDEP significantly decreased (estimated IRR = 0.4, 95% CI: 0.2–0.8, *P* = 0.008).

The rate of probable SUDEP in 2010 was pretty higher than the following year. By omitting the data of 2010, the APC of probable SUDEP rate in 2011–2019 was insignificant (APC = −2.8, 95% CI: −15.0 to 11.2, *P* = 0.6; Supplementary Figure 1). Other results including the AAPC in follow-up year (AAPC = −15.4, 95% CI: −24.9 to −4.6, *P* = 0.01; Supplementary Figure 2), IRR in the calendar year (estimated IRR = 0.9, 95% CI: 0.5–1.8, *P* = 0.8), and IRR in follow-up year (estimated IRR = 0.5, 95% CI: 0.3–0.9, *P* = 0.01) were stable. The results of APC in sensitivity analyses are shown in Supplementary Figure.

## Discussion

This is the first report to indicate that the SUDEP rate appears to decrease in calendar years and significantly decreases in a longer follow-up of the program in rural West China. Our results showed that the SUDEP occurred in 1.40 cases per thousand patient-years in convulsive epilepsy in 2010–2019 in rural western China. The sex-adjusted incidence rate of SUDEP was 1.45%0 with a slight increase.

The downward trend of the SUDEP incidence rate in our results is in agreement with the results of Ryvlin et al. (2) and Tomson et al. (3). The enrollment time of each epilepsy patient into our program was different, and patients have different times of follow-up, which may affect the rate of the SUDEP (3); therefore, we calculated the SUDEP rate with the time of follow-up. Tomson reported that the rate of SUDEP was significantly lower during the subsequent 4-year follow-up compared with the first years, with an estimated IRR of 0.75 (95% CI: 0.59–0.96) (3). The incidence of SUDEP in 2010–2014 was no significant higher than the rate in 2015–2019 in our study; however, the incidence rate of SUDEP significantly decreased during the year of management follow-up with a lower IRR. This may indicate that the management of the program plays an important role in the incidence of SUDEP. The program provided patients with drug distribution, professional medication support from a trained doctor, and SUDEP education. A recent study also emphasized the importance of medication adherence to reduce the risk of SUDEP (15). In both the calendar year and follow-up year, the crude incidence rate of SUDEP decreased over time. The reason for the downward trend may relate to attrition, natural evolution, or changes in medications or medical practice over time (2). Economic development and increased knowledge about SUDEP may also account for this finding. However, the data from Australian autopsy reports showed that several SUDEP cases were registered as other causes of death, so the mortality statistics do not tell the whole story (16).

In sensitivity analyses, after omitting the data of 2010 as an outlier, the rate of probable SUDEP decreased insignificantly in 2011–2019, but that was consistent with the result of IRR. That may give a better explanation for a different result in APC and IRR between 2010 and 2019. However, the decreased trend of SUDEP rate in the time of follow-up was stable and obviously significant when omitting the data of 2010.

Several studies have shown that SUDEP is more common in males (17, 18). However, another study found that compared with the control group, the proportion of females was greater in the SUDEP group (19). Similar to our study, the SUDEP incidence rate in females seemed higher than that in males, but the difference was not significant. This may be due to the insufficient number of SUDEP. More studies are needed to confirm this finding.

The SUDEP rate in rural western China is slightly higher than the result from a meta-analysis based on three population studies (1). The type of epilepsy was associated with the incidence rate of SUDEP (20). The patients in this program were diagnosed with generalized tonic–clonic seizures, regarded as an independent risk factor for SUDEP (21), and may have caused the overestimation of the SUDEP incidence rate in our research. On the other hand, SUDEP patients had frequent tonic–clonic seizures 3 months before their deaths (9). The SUDEP patients who died between 2010 and 2015 were partly reported in a previous study to analyze the connection between terminal seizure frequency and SUDEP (9).

The average age of this study was 41.36 years, which is consistent with the epidemiology of SUDEP. SUDEP is most common in 20- to 45-year-old patients (18). The increase in age in the program may be associated with the decreasing trend of the SUDEP incidence rate. In our studies, ~56.82% of SUDEP patients had no GTCS within 3 months before their death. Similar to the result in the North American SUDEP Registry, 42% of patients had no GTCS, 14% of patients had one GTCS 3 months before death, and 3% of patients never had a GTCS before SUDEP (22). Therefore, although GTCS is consistently considered the leading risk factor for SUDEP (23), patients with a good response to treatment and well-controlled seizures still have a great risk for SUDEP (22, 24). Because of the relative lack of tolerance or adaptive recovery responses, long periods of seizure freedom may potentially increase SUDEP risk (22).

Our study still has several limitations. First, the definition of SUDEP is probable SUDEP rather than definite SUDEP without a physical autopsy in our study. Because the population of this study was in rural areas with poor medical resources, our study was retrospective. Second, the patients of this program had convulsive epilepsy, and it was difficult to categorize the seizure types according to the 2017 classification of seizure types (25). The classification of patients in this article may not be accurate. We did not report the SUDEP rate in other types of epilepsy. Third, we had no control group to analyze the effectiveness of the program intervention for the SUDEP incidence rate. However, most epilepsy patients were enrolled in this program, so it is difficult to acquire the data of other SUDEP patients in the same area. On the other hand, we did not have enough SUDEP patients for each age group to analyze the age-adjusted incidence rate. Fourth, we calculated the crude rate of SUDEP rather than the standardized mortality rate that may have affected the results. Finally, the result in the trend of SUDEP rate in 2010–2019 was influenced much by the data of 2010. That may cause some bias because of the limited sample size. Data from retrospective interviews were also limited by recall bias.

## Conclusions

In 2010–2019, the crude incidence rate of SUDEP was 1.40% per person-year in convulsive epilepsy in rural west China. The rate seemed to present a significant downward trend with the management of the program, especially in the 5th year of follow-up. The role of program management still needs a control group to confirm this finding.

## Data Availability Statement

The raw data supporting the conclusions of this article will be made available by the authors, without undue reservation.

## Ethics Statement

The studies involving human participants were reviewed and approved by Sichuan University Ethical Standards Committee on Human Experimentation. The patients/participants provided their written informed consent to participate in this study.

## Author Contributions

HW was in charge of conceptualization, formal analysis, and writing-original draft. DC gave supporting of analysis and methodology. JH and YH were responsible for data curation equally. LL and DZ were in charge of reviewing and revising the manuscript. All authors contributed to the article and approved the submitted version.

## Funding

This work was supported by the Chinese Ministry of Health (ZX2021001) and the Sichuan Province Health Bureau (ZX2020012).

## Conflict of Interest

The authors declare that the research was conducted in the absence of any commercial or financial relationships that could be construed as a potential conflict of interest.

## Publisher's Note

All claims expressed in this article are solely those of the authors and do not necessarily represent those of their affiliated organizations, or those of the publisher, the editors and the reviewers. Any product that may be evaluated in this article, or claim that may be made by its manufacturer, is not guaranteed or endorsed by the publisher.
