# Supplementary material for: Trend of Sudden Unexpected Death in Epilepsy Incidence Rate in Rural West China
Source: Front Neurol. 2021 Sep 24;12:735201. doi: 10.3389/fneur.2021.735201 (PMC8498108; doi:10.3389/fneur.2021.735201)
Supplement: Supplementary file 1 [file Data_Sheet_1.docx]

Supplementary Material


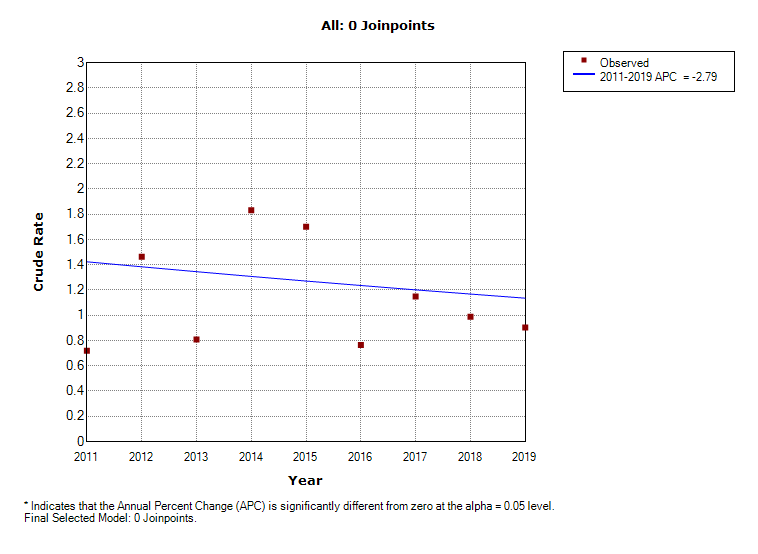


**Supplementary Figure 1.** **The APC of crude SUDEP incidence rate in 2011-2019 after omitting the data of 2010 in sensitivity analysis**


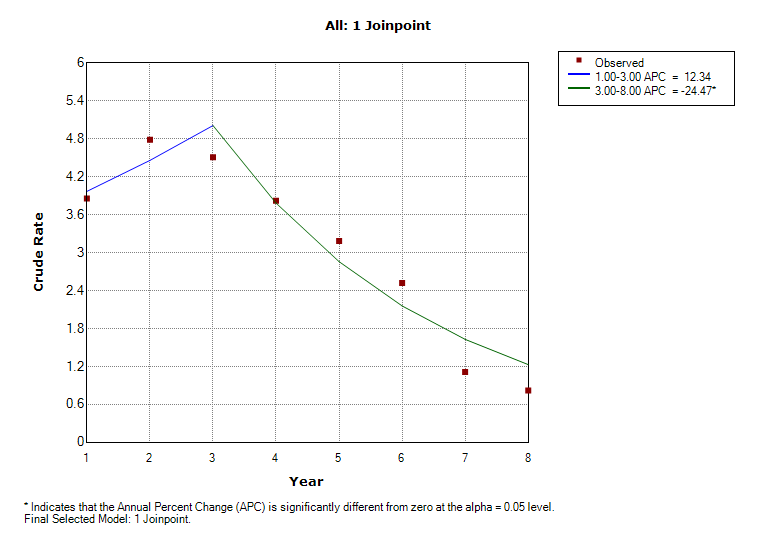


**Supplementary Figure 2.** **The APC of crude SUDEP incidence rate after 1 – 8 years of follow – up after omitting the data of 2010 in sensitivity analysis**
